# Imbalanced Angiogenesis in Pregnancies Complicated by SARS-CoV-2 Infection

**DOI:** 10.3390/v14102207

**Published:** 2022-10-07

**Authors:** Valentina Giardini, Sara Ornaghi, Carlo Gambacorti-Passerini, Marco Casati, Andrea Carrer, Eleonora Acampora, Maria Viola Vasarri, Francesca Arienti, Patrizia Vergani

**Affiliations:** 1 Department of Obstetrics and Gynecology, MBBM Foundation Onlus, San Gerardo Hospital, University of Milano-Bicocca, 20900 Monza, Italy; 2 Hematology Division, ASST-Monza, San Gerardo Hospital, University of Milano-Bicocca, 20900 Monza, Italy; 3 Laboratory Medicine, ASST-Monza, San Gerardo Hospital, University of Milano-Bicocca, 20900 Monza, Italy

**Keywords:** COVID-19, SARS-CoV-2, angiogenic factors, sFlt-1, endothelial dysfunction, preeclampsia, placental dysfunction

## Abstract

COVID-19 and preeclampsia (preE) share the ANG-II mediated endothelial dysfunction, resulting from a significant dysregulation of RAS and an imbalanced proportion of anti-angiogenic and pro-angiogenic soluble plasmatic factors. Of note, an increased incidence of preE has been reported among COVID-19-infected mothers compared to the general pregnant population. The two most promising angiogenic markers are the soluble fms-like tyrosine kinase receptor-1 (sFlt-1), the major antiangiogenic factor, and the placental growth factor (PlGF), a powerful angiogenic factor. Since these markers have proven useful in the prediction, diagnosis, and severity of preE, this study aimed to evaluate their maternal serum levels in pregnancies complicated by SARS-CoV-2 infection and to assess their potential use to guide the management of these women. A retrospective analysis of SARS-CoV-2-positive pregnant women was performed. The serum levels of sFlt-1 and PlGF were collected at the diagnosis of SARS-CoV-2 infection at the hospital, before the beginning of steroid/hydroxychloroquine and/or antithrombotic therapy. The sFlt-1/PlGF ratio was stratified using cut-off values clinically utilized in the diagnosis and prediction of preE (low < 38, intermediate 38–85/110* and high >85/110*, * if before or after the 34th week of gestation). A total of 57 women were included, of whom 20 (35%) had signs and symptoms of COVID-19 at hospital presentation and 37 (65%) were asymptomatic. None were vaccinated. The mean gestational age at diagnosis of SARS-CoV-2 infection was 32 weeks in symptomatic patients and 37 weeks and 5 days in asymptomatic ones (*p* = 0.089). sFlt-1 serum levels were higher in SARS-CoV-2 positive asymptomatic patients compared to women with COVID-19 related symptoms (4899 ± 4357 pg/mL vs. 3187 ± 2426 pg/mL, *p* = 0.005). sFlt-1/PlGF at admission was <38 in 18 of the 20 symptomatic women (90%) compared to 22 (59%) of the asymptomatic patients (*p* = 0.018). Of note, two of the three women admitted to the intensive care unit had a very low ratio (<2). In turn, rates of patients with sFlt-1/PlGF at admission > 85/110 were not significantly different between the two groups: 11% in asymptomatic patients (4/37) vs. none of the symptomatic patients (*p* = 0.286), and all of them presented a placental dysfunction, like preE (*n* = 1) and FGR (*n* = 3). Of note, there were no stillbirths or maternal or neonatal deaths among symptomatic patients; also, no cases of preE, FGR, or small for gestational age neonates were diagnosed. In conclusion, our data suggest that SARS-CoV-2 infection during pregnancy could influence the angiogenic balance. A significant pathological alteration of the sFlt-1/PlGF ratio cannot be identified during the symptomatic phase; however, if left untreated, SARS-CoV-2 infection could potentially trigger placental dysfunction.

## 1. Introduction

COVID-19 is a pandemic infection caused by the severe acute respiratory syndrome-coronavirus-2 (SARS-CoV-2), a single-stranded RNA coronavirus [1,2].

SARS-CoV-2 primarily infects the respiratory system but may also damage other organs and tissues, displaying the receptor used by the virus for cell entry, the angiotensin-converting enzyme 2 (ACE2) [3]. 

ACE2 is an enzyme of the renin-angiotensin system (RAS), a hormone signaling cascade regulating blood pressure and fluid in the body. In the case of impaired renal perfusion, renin activates a series of enzymatic reactions that lead to the formation of angiotensin II (ANG II), a vasoconstrictor, also involved in inflammation, fibrosis, coagulation activation, and immune response. ACE2 converts ANG II to angiotensin 1–7 (ANG 1–7), a vasodilator that counteracts the effects of ANG II [4]. 

Lung tissue has high RAS activity, and it is the main site of ANG II synthesis. During SARS-CoV-2 infection, ACE2 downregulation occurs due to the use of ACE2 by the virus for cell entry. Such downregulation leads to reduced ANG 1-7 formation and, ultimately, to ANG II-mediated endothelial dysfunction [5]. 

Alongside the lung, the placenta is another major extra-renal RAS activity site. Placental RAS has been reported to play an important role in the pathogenesis of preeclampsia (preE), a multisystem, pregnancy-specific disorder [6,7].

RAS components regulate angiogenesis, which is important for vascular homeostasis and placentation [8,9]. preE is characterized by an imbalance between pro-angiogenic factors, such as vascular endothelial growth factor (VEGF) and placental growth factor (PlGF), and anti-angiogenic factors, such as soluble fms-like tyrosine kinase 1 (sFlt-1). sFlt1 antagonizes VEGF and PlGF in circulation by binding and preventing their interaction with their endothelial receptors, thus determining endothelial dysfunction [10,11]. sFlt-1 production is directly related to the degree of placental hypoxia by means of placental ANG II activity [8]. 

A few recent studies have highlighted a possible link between COVID-19 and preE. Mendoza and colleagues have shown that a preE-like syndrome can be induced by severe COVID-19 in pregnant women [12]. In addition, an increased incidence of preE has been reported among mothers infected with SARS-CoV-2 compared to the general obstetric population [13,14]. Of note, both COVID-19 and preE display a significant dysregulation of RAS, which leads to ANG-II-mediated endothelial dysfunction. 

Our group showed for the first time that SARS-CoV-2-infected non-pregnant individuals with pneumonia have an angiogenic imbalance like preE: the levels of sFlt-1 were significantly higher in patients with SARS-CoV-2 pneumonia compared to patients with pneumonia due to other causes or with healthy controls. PlGF values were not significantly affected by COVID-19, but the sFlt1/PlGF ratio was higher in COVID-19-positive than in COVID-19-negative pneumonia cases [15]. Subsequently, other authors have confirmed the increased sFlt-1 levels in severe COVID-19 patients and identified sFlt-1 as a biomarker to predict survival and thrombotic events in COVID-19 individuals [16]. 

However, the role of the angiogenic markers sFlt1 and PlGF in pregnancies complicated by SARS-CoV-2 infection is still unclear [17]. 

Recently, Espino-y-Sosa and colleagues have shown that high levels of sFlt-1/ANG-II are associated with adverse outcomes, including severe pneumonia, intensive care unit (ICU) admission, intubation, viral sepsis, and death, among SARS-CoV-2-infected pregnant women [18]. Subsequently, the same group has identified higher values of sFlt-1 MoM in pregnant women with severe COVID-19 and that these values could be used as a predictor of medical complications, similarly to sFlt-1/ANG-II [19].

Here we investigate the maternal serum levels of sFlt-1 and PlGF in pregnancies complicated by SARS-CoV-2 infection and assess the potential relation between such levels and adverse obstetric outcomes related to endothelial dysfunction, including preE and fetal growth restriction (FGR).

## 2. Materials and Methods

A retrospective analysis including all positive SARS-CoV-2 pregnant women was performed at the Obstetrics Unit of Fondazione MBBM Onlus at San Gerardo Hospital in Monza, Italy from April 2020 to October 2021. The study was approved by our Ethics Committee and conducted according to the Declaration of Helsinki principles.

The primary outcome was to assess the angiogenic profile in pregnant patients with SARS-CoV-2 infection with respect to the course of infection (symptomatic vs. asymptomatic) and its relationship with respiratory and obstetric complications. The secondary outcome was the analysis of additional laboratory tests comparing symptomatic to asymptomatic women.

Patients already on drug therapy (enoxaparin sodium, steroids, or hydroxychloroquine) as well as patients without a chest X-ray performed at the time of hospital admission were excluded. 

The pregnant women who tested positive for SARS-CoV-2 infection were divided into two groups: women with signs and symptoms of COVID-19 at the time of hospital presentation (symptomatic group) and asymptomatic women. 

Signs and symptoms of COVID-19 included respiratory symptoms such as fever, cough, shortness of breath, rhinitis, but also pharyngodynia, fatigue, muscle or body aches, arthralgia, ageusia, and anosmia.

Blood samples were obtained upon diagnosis of SARS-CoV-2 infection by RT-PCR assay on nasopharyngeal swabs before the beginning of any therapy. 

sFlt-1 and PlGF were measured on a Roche Cobas e601 platform using the electrochemiluminescence immunoassay principle (REF 05109523190 and 05144671190, respectively). Table 1 displays normal values of sFlt-1, PlGF, and the sFlt-1/PlGF ratio based on the analysis of a total of 877 European women with a singleton pregnancy and a normal pregnancy outcome, obtained in the Prospective Multicenter Study: Diagnosis of Preeclampsia by means of the Elecsys sFlt-1 assay and the Elecsys PlGF assay (Roche study No. CIM RD000556/X06P006) [20].

sFlt-1/PlGF ratio was stratified using cut-off values clinically utilized in the diagnosis and prediction of preE (low < 38, intermediate 38–85/110, and high > 85/110) and analyzed based on the outcome of the pregnancy. sFlt-1/PlGF ratios of >85 (20 to 33 weeks and 6 days) and >110 (34 weeks to delivery) have been shown to be highly suggestive of preE [20]. The PROGNOSIS study data demonstrated the ability of the sFlt-1/PlGF ratio cut-off of 38 to predict a combined endpoint of preE, eclampsia, or HELLP syndrome or maternal or fetal negative outcomes [21].

The following laboratory tests were analyzed: leukocytes, neutrophils, lymphocytes, platelets, creatinine, uric acid, aspartate aminotransferase (AST), alanine aminotransferase (ALT), lactic dehydrogenase (LDH), partial thromboplastin time (PTT) ratio, prothrombin time (PT) ratio, fibrinogen, D-dimer, antithrombin III, N-terminal pro-B-type natriuretic peptide (NT-proBNP), total calcium, albumin, D vitamin, C-reactive protein (C-RP), and procalcitonin. C-RP was measured on a Roche Cobas c702 platform using an immunoturbidimetric method (REF 07876424190), with normal values < 5 mg/L. D-Dimer values were measured on an ACL TOP 500 platform (Instrumentation Laboratory) using a chromogenic method with normal values < 250 ng/mL.

The following data about anamnestic details and course of infection were collected from patients’ medical records: age, parity, pregestational obesity (body mass index, BMI ≥ 30 kg/m^2^), pregestational and gestational diabetes, chronic hypertension, low-dose aspirin use during pregnancy for obstetric reasons, year of infection 2020 vs. 2021, mean gestational age at first positive SARS-CoV-2 swab, number of weakly positive swabs, positivity at the surveillance swab, SARS-CoV-2 close contact exposure, mean gestational age at onset of COVID-19-related symptoms, respiratory symptoms at hospital admission, mean gestational age at diagnosis of pneumonia on chest X-ray, latency time between the onset of COVID-19 symptoms and the diagnosis of pneumonia, latency time between the onset of COVID-19 symptoms and blood tests, the requirement of high dependency/intensive care unit (ICU) admission, therapy (enoxaparin sodium, steroid, hydroxychloroquine, antibiotics), the need for oxygen supplementation, continuous positive airway pressure (CPAP), and endotracheal intubation, and cases of maternal, fetal, and neonatal death. 

The following obstetric outcomes were also recorded: cases of hypertensive disorders during pregnancy or the post-partum period, preE, FGR, premature birth < 37 weeks), latency time between the onset of COVID-19 symptoms and delivery, latency time between COVID-19 pneumonia and delivery, latency time between blood tests and delivery, delivery mode (vaginal vs. cesarean section), urgent cesarean section for maternal respiratory distress, birthweight, and small for gestational age (SGA) newborns.

preE was defined by the most recent ACOG’s clinical criteria [22]. Diagnosis of FGR was made according to the International Delphi consensus [23], while an SGA newborn was defined as a birthweight below the 10th percentile according to the Italian Neonatal Study (INeS) reference charts [24].

Discrete variables were reported as numbers and percentages, and continuous variables were reported as mean and standard deviation. 

Differences between groups were evaluated using the Wilcoxon–Mann–Whitney test or Fisher’s Exact and *p* < 0.05 was considered statistically significant.

## 3. Results

The study population, after counting the losses to follow-up (*n* = 6), included 57 SARS-CoV-2-positive pregnant women, who were divided into two groups: 20 (35%) were symptomatic whereas 37 were asymptomatic. 

Table 2 shows general maternal characteristics. We did not identify any differences between the two study groups. None of the women were vaccinated for SARS-CoV-2. 

The data on SARS-CoV-2 infection are reported in Table 3.

Seventy-five percent of the women included in the study acquired the infection in 2020, and 26% had a close-contact exposure.

The mean gestational age at diagnosis of SARS-CoV-2 infection was 37 weeks and 5 days in asymptomatic women and 32 weeks in symptomatic ones (*p* = 0.089). Asymptomatic women were identified by the surveillance swab required before hospital admission for obstetric reasons in 86% of cases (*n* = 32), and only in 16% (6/37) were we able to date infection as recent (within 10 days); nine (24%) swabs were weakly positive.

Among the 20 women with symptoms of SARS-CoV-2 infection, the mean number of days between symptoms onset and hospital access was 5 ± 4 days. SARS-CoV-2-related pneumonia was identified by chest X-ray in 80% of symptomatic women vs. 11% of those who were asymptomatic. The mean gestational age at diagnosis of radiologic pneumonia was 30 weeks and 4 days in symptomatic cases compared to 39 weeks and 5 days in asymptomatic ones (*p* = 0.035). 

Seven (35%) symptomatic women required high-dependency/intensive care. Precisely two of the six women admitted to a semi-intensive ward were transferred to ICU during hospitalization, and one was admitted to the ICU immediately after the cesarean section was performed for respiratory failure. None of the asymptomatic patients needed high-dependency/intensive care during hospitalization.

All women received therapy with enoxaparin sodium until a negative naso-pharyngeal swab or until the end of pregnancy if risk factors were present; 14 (70%) were given steroids, and 2 (10%) hydroxychloroquine (first pandemic wave). Eight (40%) patients also received antibiotic therapy. A total of 14 (70%) women needed oxygen supplementation, while a continuous positive airway pressure was applied in 5 cases (25%) and 3 (15%) underwent endotracheal intubation (2 before the cesarean section and one at 23 weeks and 6 days). 

Table 4 shows obstetric outcomes comparing symptomatic vs. asymptomatic women at hospital admission. 

There were no significant differences between the two groups in the mean gestational age at delivery, the mode of delivery, or the labor induction rate. The mean gestational age at delivery in the asymptomatic women was 39 weeks and 2 days while in the symptomatic ones it was 38 weeks and 2 days with a mean latency time between symptom onset and delivery of 48 ± 44 days and 52 ± 44 days from the diagnosis of pneumonia. All asymptomatic patients delivered at term, while four (20%) symptomatic ones had a preterm delivery before 37 weeks (*p* = 0.012): two patients for respiratory failure at 36 weeks and 5 days and at 30 weeks and 2 days, respectively, one woman after premature rupture of membranes at 35 weeks and 3 days, and the remaining one for placenta previa. Eight (40%) symptomatic women delivered by cesarean section, but only in two (10%) cases for respiratory failure. 

There were no cases of preE, FGR, or SGA neonates among symptomatic women. In turn, in asymptomatic women, there were four (11%) cases of hypertensive disorder and three cases of FGR, two of which were also identified as SGA neonates at birth. In addition, four other SGA newborns were also reported in this group, three of which were not known in the prenatal period. The mean birthweight did not differ in the two groups.

Table 5 shows blood tests performed at hospital access in the study population. We found higher sFlt-1 serum levels in asymptomatic patients compared to symptomatic ones (sFlt-1: 4899 ± 4357 vs. 3187 ± 2426 pg/mL, *p* = 0.005).

The mean sFlt-1/PlGF was 50 ± 58 in the asymptomatic group and 17 ± 23 in the symptomatic one (*p* = 0.099). sFlt-1/PlGF at admission was <38 (i.e., low risk) in 18 of the 20 symptomatic women compared to 22 (59%) of the asymptomatic ones (*p* = 0.018). In turn, rates of patients with sFlt-1/PlGF at admission >85/110 (i.e., high risk) were not significantly different between the two groups: 11% in the asymptomatic (4/37) vs. none in the symptomatic group (*p* = 0.286). Of note, all four asymptomatic women with a high-risk sFlt-1/PlGF ratio displayed a condition of placental dysfunction, such as preE (*n* = 1) or FGR (*n* = 3). The patient with preE without severity criteria at 38 weeks and 6 days, was a 38-year-old primigravida in good health, who reported having COVID-19 at 23 weeks; she was induced and vaginally delivered a newborn with an appropriate weight for its gestational age. Two of the three FGRs were diagnosed at the term of pregnancy (38 weeks and 6 days—41 weeks and 3 days). One FGR case was associated with oligohydramnios, whereas another one was an early-onset case (diagnosed at 22 weeks) delivered by cesarean section at 30 weeks and 2 days. All three women with a growth-restricted fetus did not complain of COVID-19-related symptoms in the weeks prior to the positive surveillance swab. All pregnant women with an SGA newborn at birth, who was not FGR in the prenatal period, showed a normal sFlt-1/PlGF ratio < 38.

In the seven patients that required high-dependency/intensive care, the mean sFlt-1/PlGF was 18 ± 30, with a sFlt-1 mean of 3225 ± 2966 pg/mL and a PlGF mean of 490 ± 325 pg/mL. Two of the three patients admitted to the ICU displayed very low values of sFlt-1/PlGF ratio: 1.2 and 1.67. 

Regarding other laboratory tests, women with symptoms had higher levels of C-RP compared to asymptomatic patients (0.96 ± 1.3 vs. 4.9 ± 4.3 mg/dL, *p* = 0.018).

## 4. Discussion

As evidenced by a recent literature review, preE is one of the “great obstetrical syndromes” with multiple etiologic factors that converge to cause endothelial cell dysfunction, intravascular inflammation, and syncytiotrophoblast stress. COVID-19 is among the aetiologies cited [25]. Indeed, preE and COVID-19 have common pathogenic pathways [26]. Both diseases are characterized by significant alterations in the RAS with an imbalanced proportion of anti-angiogenic and pro-angiogenic soluble plasmatic factors [15]. Of note, an increased incidence of preE has been reported among COVID-19-infected mothers compared to the general pregnant population [8,14,27].

The presence of an angiogenic imbalance is important not only for gaining a better understanding of the pathogenesis of these two diseases but also for clinical purposes. By dosing sFlt-1 and PlGF, both angiogenic biomarkers, it is possible to evaluate the severity of preE [28] as well as of COVID-19 in non-pregnant patients [15,16]. 

The role of the angiogenic markers in pregnancies complicated by SARS-CoV-2 infection is unclear due to the interference of the placenta, one of the major extrarenal RAS production sites and the main source of circulating antiangiogenic factors during pregnancy [6,17].

Mendoza et al. showed that pregnant women with severe COVID-19 can develop a preE-like syndrome that might be distinguished from actual preE by assessing the sFlt-1/PlGF ratio [12]. 

Recently Espino-y-Sosa and colleagues reported that the sFlt-1/ANG-II ratio could be a good predictor of adverse outcomes among pregnant women with COVID-19, including pneumonia, ICU admission, intubation, viral sepsis, and death, confirming the role of the RAS system in the pathogenesis of COVID-19 [18]. This work has demonstrated that women with severe COVID-19 pneumonia have higher levels of sFlt-1 and a higher sFlt-1/PlGF ratio and lower plasma levels of ANG-II. PlGF values were not significantly different between severe and non-severe pregnant cases of COVID-19 pneumonia. A subsequent publication by the same group has shown that sFlt-1 MoM values are higher in pregnant women with severe COVID-19 and they can be used as a predictor of medical complications, similarly to sFlt-1/ANG-II [19]. 

Based on this small cohort, our data suggest that SARS-CoV-2 infection during pregnancy could influence the angiogenic profile, but likely with a different effect according to the type of viral infection, symptomatic vs. asymptomatic. Unlike Espino-y-Sosa et al. and Torres-Torres et al. [18,19], who included only symptomatic women later divided into severe and non-severe COVID-19, we differentiated the patients into symptomatic and asymptomatic for SARS-CoV-2 infection.

We identified higher sFlt-1 serum levels in SARS-CoV-2 positive asymptomatic patients compared to women with SARS-CoV-2-related symptoms. Moreover, a substantially lower rate of asymptomatic women displayed low-risk sFlt-1/PlGF ratio values at hospital admission compared to symptomatic women (59% vs. 90%, *p* = 0.018). In turn, rates of patients with high-risk sFlt-1/PlGF ratio values were not significantly different between asymptomatic and symptomatic patients, and all four asymptomatic women with high-risk values showed a placental dysfunction-related obstetric condition, including preE (*n* = 1) and FGR (*n* = 3). Interestingly, women with an SGA newborn with no antenatal FGR or with hypertensive disorders other than preE had a normal sFlt-1/PlGF ratio value. These findings suggest that sFlt-1 and PlGF might be useful for the differential diagnosis of conditions related to altered placental function. 

In their recent papers, Espino-y-Sosa et al. and Torres-Torres et al. have reported worse maternal and neonatal outcomes compared to us, as well as different values in the angiogenic biomarkers analyzed [18,19]. Possible explanations, as we stated in a letter to the editor [29], may include the following: (1) a delayed hospital admission of severe COVID-19 patients, leading to increased sFlt-1 levels due to prolonged exposure to hypoxia, whereas the mean number of days between symptom onset and hospital admission in our symptomatic women was 5; (2) the dosage of sFlt-1 and PlGF was taken from plasma obtained at hospital admission and stored at −70 °C until analysis, whereas we dosed the biomarkers on serum samples as soon as they were collected with no previous storage, as per Roche Diagnostics instructions; (3) a later gestational period at sFlt-1 assessment in our asymptomatic patients; and (4) a potential influence of pre-hospital treatment on biomarker levels in the women included in [18,19], although no reference to such treatment is available in either of the studies. 

Contrasting data are available in the literature regarding the association between SARS-CoV-2 infection in pregnancy and placental dysfunction. A potential explanation might be related to the latency between symptom onset-delivery and the administered treatment. Additionally, SARS-CoV-2 infection can induce a preE-like syndrome not related to placental dysfunction. Thus, the sFlt-1/PlGF ratio values could be used for differential diagnosis. No significant differences in the incidence of preE were identified between asymptomatic and symptomatic patients in our cohort. Furthermore, no cases of preE, FGR, or SGA neonates were diagnosed among symptomatic patients. Considering the mean latency time of 48 days between COVID-19 symptoms and delivery, it would also be interesting to investigate the role of therapy, particularly using enoxaparin sodium, in the prevention of preE and FGR. Moreover, McLaughlin et al. have recently demonstrated that low-molecular-weight heparin can acutely improve endothelium-dependent relaxation in pregnant women at high risk of severe preE and significantly increase circulating maternal levels of PlGF [30].

A recent sub-analysis from the INTERCOVID study population has shown that COVID-19 during pregnancy is independently associated with preE. Interestingly, this association is not modified by COVID-19 severity [31]. The sFlt-1/PlGF results and obstetric outcomes among SARS-CoV-2-infected asymptomatic women are of particular interest because they did not receive a specific therapy for viral infection and often the infection cannot be dated. If the increase in sFlt-1 levels we observed is the consequence of viral infection, and not just of the higher gestational age at its evaluation, then the identification of these women might be important since they may benefit from more intensive antenatal surveillance of fetal growth and blood pressure due to a potential increased risk of placental dysfunction.

As for the diagnosis of pneumonia, the distinction between clinical and radiological pneumonia is very important. In particular, COVID-19 radiological pneumonia was found in 4/37 asymptomatic women (11%) vs. 16/20 (80%) in the symptomatic group. Therefore, this aspect deserves further investigation regarding the long-term effects and the need for future follow-up of these patients.

Several studies have reported an increased risk of preterm delivery in pregnant women with symptomatic SARS-CoV-2 infection [32,33]. In line with these findings, we observed preterm birth before 37 weeks only in symptomatically infected women, whereas all asymptomatic cases gave birth at term. 

Regarding laboratory exams other than angiogenic biomarkers, women with symptoms displayed higher levels of C-RP. This result mirrors that reported by Espino-y-Sosa and Torres-Torres and colleagues [18,19]. Thus, this laboratory test might be useful for dating the viral infection and for defining the severity of the clinical condition.

## 5. Conclusions

In conclusion, our data show that SARS-CoV-2 infection during pregnancy could influence the angiogenic balance, especially in asymptomatic women. 

COVID-19 and preE are vascular diseases that begin in the lungs and in the placenta, respectively, and both end in the endothelium. They share an ANG-II mediated endothelial dysfunction secondary to an angiogenic imbalance, which in turn can lead to preE and/or FGR. We believe that the interpretation of the obstetric outcomes in COVID-19-infected pregnant women should consider the type of infection (symptomatic vs. asymptomatic), the latency between infection and delivery, and the administered therapy performed. 

This study could clarify some etiopathogenetic aspects of SARS-CoV-2 infection in pregnancy and the role of timely and adequate therapy to prevent both obstetric and non-obstetric complications. In addition, our study suggests that abnormally high sFlt-1/PlGF ratio values could help in detecting placental dysfunction even in SARS-CoV-2- positive pregnant women. 

In view of the small sample size, these preliminary observations need to be confirmed by large-scale studies. Furthermore, future studies should focus on providing more in-depth details of the angiogenic profile during SARS-CoV-2 infection in both pregnant and non-pregnant individuals. 

## Figures and Tables

**Table 1:** Serum values of sFlt-1 (pg/mL), PlGF (pg/mL), and sFlt-1/PlGF ratio in patients with a normal singleton pregnancy. The median as well as the centiles (Q) 10 and 90 are shown—data from Roche study No. CIM RD000556/X06P006 [20].

	Completed Weeks of Gestation						
**Centiles**	**10–14**	**15–19**	**20–23**	**24–28**	**29–33**	**34–36**	**≥37**
**sFlt-1**							
Q 10	776	844	718	722	967	1220	1899
Median	1328	1355	1299	1355	1742	2552	3485
Q 90	2174	2453	2605	2557	3650	5620	7901
**PlGF**							
Q 10	31.3	80.9	143	200	139	98.2	68.6
Median	52.6	135	264	465	471	284	191
Q 90	100	251	500	921	1073	831	620
**sFlt-1/PlGF ratio**							
Q 10	11.6	4.67	2.22	1.22	1.22	2.15	3.81
Median	24.8	10.5	4.92	3.06	3.75	9.03	19.6
Q 90	46.6	20.5	11.0	7.49	16.1	43.4	85.7

**Table 2:** Population characteristics comparing positive SARS-CoV-2 pregnant women with signs and symptoms of COVID-19 at the hospital admission vs. asymptomatic women [Mean; standard deviation—SD; *n*; (%)].

**SARS-CoV-2 Pregnant Women**	**Asymptomatic**	**Symptomatic**	***p* Value**
*n*—%	37 (65)	20 (35)	
**Variables**			
Age (years)	33 ± 5	33 ± 4	0.667
Italian	20 (54)	10 (50)	0.788
Nulliparous	14 (38)	6 (30)	0.772
Obesity (BMI ≥ 30 kg/m^2^)	7 (19)	5 (25)	0.736
Diabetes/gestational diabetes mellitus	10 (27)	5 (25)	0.580
Chronic hypertension	2 (5)	0 (0)	0.536
Low-dose Aspirin use during pregnancy	6 (16)	2 (10)	0.699

Legend: BMI = body mass index.

**Table 3:** SARS-CoV-2 infection characteristics comparing symptomatic vs. asymptomatic women at the hospital admission [Mean; standard deviation—SD; *n*; (%)].

**SARS-CoV-2 Pregnant Women**	**Asymptomatic**	**Symptomatic**	***p* Value**
*n*—%	37 (65)	20 (35)	
**Variables**			
Infection in 2020	34 (92)	9 (45)	0.0001
Infection in 2021	3 (8)	11 (55)	
GA at positive swab (weeks.days ± weeks)	37.5 ± 5	32 ± 6	0.089
Weakly positive swab	9 (24)	0 (0)	0.020
Positivity at the surveillance swab	32 (86)	0 (0)	0.0001
SARS-CoV-2 close contact exposure	9 (24)	6 (30)	0.755
GA at COVID-19 symptoms onset (weeks.days ± weeks)	33.4 ± 7	31.3 ± 6	0.873
Respiratory symptoms at admission	0 (0)	16 (80)	0.0001
Pneumonia on chest X-ray	4 (11)	16 (80)	0.0001
GA at diagnosis of pneumonia on chest X-ray (weeks.days ± weeks)	39.5 ± 1	30.4 ± 6	0.035
Latency time between COVID-19 symptoms—pneumonia (days)	11 ± 5	6 ± 3	NA
High dependency unit admission	0 (0)	6 (30)	0.001
ICU admission	0 (0)	3 (15)	0.039
Enoxaparin sodium therapy	37 (100)	20 (100)	1.000
Steroid therapy	0 (0)	14 (70)	0.0001
Hydroxychloroquine therapy	3 (8)	2 (10)	1.000
Antibiotic therapy	1 (3)	8 (40)	0.001
Oxygen supplementation	0 (0)	14 (70)	0.0001
Continuous positive airway pressure (CPAP)	0 (0)	5 (25)	0.004
Intubation	0 (0)	3 (15)	0.039
Maternal/fetal/neonatal death	0 (0)	0 (0)	NA

Legend: GA = gestational age; ICU = intensive care unit.

**Table 4:** Obstetric outcomes comparing symptomatic vs. asymptomatic women at the hospital admission [Mean; standard deviation—SD; *n*; (%)].

**SARS-CoV-2 Pregnant Women**	**Asymptomatic**	**Symptomatic**	***p* Value**
*n*—%	37 (65)	20 (35)	
**Variables**			
Hypertensive disorders in pregnancy/post-partum	4 (11)	1 (5)	0.647
Preeclampsia (preE)	2 (5)	0 (0)	0.536
Fetal growth restriction (FGR)	3 (8)	0	0.545
Premature birth < 37 weeks	0 (0)	4 (20)	0.012
GA at delivery (weeks.days ± weeks)	39.2 ± 2	38.2 ± 2	0.704
Latency time between COVID-19 symptoms—delivery (days)		48 ± 44	
Latency time between COVID-19 pneumonia—delivery (days)		52 ± 44	
Latency time between blood tests—delivery (days)	6 ± 21	43 ± 43	0.041
Vaginal delivery	23 (62)	12 (60)	1.000
Cesarean section	14 (38)	8 (40)	1.000
Urgent cesarean section for maternal respiratory distress	0 (0)	2 (10)	0.119
Induced labor	18 (49)	6 (30)	0.261
RDS prophylaxis for COVID-19	0 (0)	3 (15)	0.039
Birthweight (grams)	3205 ± 649	3070 ± 459	0.373
Small for gestational age (SGA)	6 (16)	0 (0)	0.081

Legend: GA = gestational age; RDS = respiratory distress syndrome; Fetal growth restriction (FGR): International Delphi consensus [23]; Small for gestational age newborn (SGA): birthweight below the 10th percentile (INeS charts) [24].

**Table 5:** Blood tests performed at the hospital access in the study population, comparing symptomatic vs. asymptomatic women [Mean; standard deviation—SD; *n*; (%)].

**SARS-CoV-2 Pregnant Women**	**Asymptomatic**	**Symptomatic**	***p* Value**
*n*—%	37 (65)	20 (35)	
**Variables**			
GA at blood tests (weeks.days ± weeks)	38.3 ± 4	32.1 ± 6	0.208
Latency time between COVID-19 symptoms—blood tests (days)	27 ± 34	5 ± 4	0.038
Angiogenic factors			
sFlt-1 (pg/mL)	4899 ± 4357	3187 ± 2426	0.005
PlGF (pg/mL)	178 ± 104	346 ± 232	0.099
sFlt1/PlGF	50 ± 58	17 ± 23	0.099
sFlt1/PlGF < 38	22 (59)	18 (90)	0.018
sFlt1/PlGF 38-85/110 * (* after 34 weeks)	11 (30)	2 (10)	0.111
sFlt1/PlGF > 85/110 * (* after 34 weeks)	4 (11)	0 (0)	0.286
Other laboratory tests			
Leukocytes (×10^3^/μL)	9.2 ± 2.5	8.4 ± 3.3	0.889
Neutrophils (×10^3^/μL)	8.7 ± 12.8	6.7 ± 3.0	0.795
Lymphocytes (×10^3^/μL)	1.8 ± 0.6	1.5 ± 1.5	0.542
Platelets (×10^3^/μL)	217 ± 65	190 ± 48	0.119
Creatinine (mg/dL)	0.7 ± 0.1	0.7 ± 0.1	0.920
Uric acid (mg/dL)	4.7 ± 1.3	4.1 ± 1.3	0.764
AST (U/L)	24 ± 15	30 ± 22	0.529
ALT (U/L)	27 ± 36	23 ± 20	0.857
LDH (U/L)	195 ± 45	204 ± 57	0.889
PTT (ratio)	0.87 ± 0.08	0.96 ± 0.08	0.317
PT (ratio)	0.93 ± 0.07	0.95 ± 0.06	0.719
Fibrinogen (mg/dL)	469 ± 115	480 ± 78	0.147
D-dimer (ng/mL)	801 ± 663	621 ± 479	0.156
Antithrombin III (%)	98 ± 18	95 ± 22	0.441
NT-proBNP	63 ± 53	97 ± 132	0.928
Total calcium (mg/dL)	8.8 ± 0.4	8.4 ± 0.3	0.719
Albumin (g/dL)	3.5 ± 0.3	3.3 ± 0.2	0.171
D Vitamin (ng/mL)	20.0 ± 12.2	19.3 ± 11.2	0.412
C-RP (mg/dL)	0.96 ± 1.3	4.9 ± 4.3	0.018
Procalcitonin (ng/mL)	0.08 ± 0.08	0.21 ± 0.31	0.242

Legend: GA = gestational age; sFlt-1 = soluble fms-like tyrosine kinase 1; PlGF = placental growth factor * The high cut-off value of the sFlt-1/PlGF ratio is <85 before 34 weeks and <110 after 34 weeks. AST = aspartate aminotransferase; ALT = alanine aminotransferase; LDH = lactate dehydrogenase; PTT = partial thromboplastin time; PT = prothrombin time; NT-pro BNP = N-terminal prohormone of brain natriuretic peptide; C-RP = C-reactive protein.
